# Medical and nursing students' intentions to work abroad or in rural areas: a cross-sectional survey in Asia and Africa

**DOI:** 10.2471/BLT.14.136051

**Published:** 2014-09-03

**Authors:** David M Silvestri, Meridith Blevins, Arfan R Afzal, Ben Andrews, Miliard Derbew, Simran Kaur, Mwapatsa Mipando, Charles A Mkony, Philip M Mwachaka, Nirju Ranjit, Sten Vermund

**Affiliations:** a Vanderbilt University Institute for Global Health, Vanderbilt University School of Medicine, 2525 West End Ave, Suite 750, Nashville, Tennessee, 37203, United States of America (USA).; b International Centre for Diarrhoeal Disease Research, Dhaka, Bangladesh.; c Department of Internal Medicine, University of Zambia School of Medicine, Lusaka, Zambia.; d Department of Surgery, Addis Ababa University School of Medicine, Addis Ababa, Ethiopia.; e Department of Physiology, Maulana Azad Medical College, New Delhi, India.; f University of Malawi College of Medicine, Blantyre, Malawi.; g Department of Surgery, Muhimbili University of Health and Allied Sciences, Dar es Salaam, United Republic of Tanzania.; h Department of Human Anatomy, University of Nairobi, Nairobi, Kenya.; i Department of Anatomy, Tribhuvan University Institute of Medicine, Kathmandu, Nepal.

## Abstract

**Objective:**

To assess medical and nursing students’ intentions to migrate abroad or practice in rural areas.

**Methods:**

We surveyed 3199 first- and final-year medical and nursing students at 16 premier government institutions in Bangladesh, Ethiopia, India, Kenya, Malawi, Nepal, the United Republic of Tanzania and Zambia. The survey contained questions to identify factors that could predict students’ intentions to migrate. Primary outcomes were the likelihoods of migrating to work abroad or working in rural areas in the country of training within five years post-training. We assessed predictors of migration intentions using multivariable proportional odds models.

**Findings:**

Among respondents, 28% (870/3156) expected to migrate abroad, while only 18% (575/3158) anticipated a rural career. More nursing than medical students desired professions abroad (odds ratio, OR: 1.76; 95% confidence interval, CI: 1.25–2.48). Career desires before matriculation correlated with current intentions for international (OR: 4.49; 95% CI: 3.21–6.29) and rural (OR: 4.84; 95% CI: 3.52–6.66) careers. Time spent in rural areas before matriculation predicted the preference for a rural career (20 versus 0 years: OR: 1.53, 95% CI: 1.19–1.98) and against work abroad (20 versus 0 years: OR: 0.69, 95% CI: 0.50–0.96).

**Conclusion:**

A significant proportion of students surveyed still intend to work abroad or in cities after training. These intentions could be identified even before matriculation. Admissions standards that account for years spent in rural areas could promote greater graduate retention in the country of training and in rural areas.

## Résumé

**Objectif:**

Évaluer les intentions des étudiants en médecine et en soins infirmiers concernant une émigration à l'étranger ou une pratique dans les zones rurales.

**Méthodes:**

Nous avons interrogé 3199 étudiants en première année et en dernière année de médecine ou de soins infirmiers dans les 16 plus grandes institutions gouvernementales au Bangladesh, en Ethiopie, en Inde, au Kenya, au Malawi, au Népal, en Tanzanie et en Zambie. L'enquête contenait des questions pour identifier les facteurs qui pourraient prédire les intentions d'émigration des étudiants. Les résultats primaires étaient les probabilités d'émigration en vue de travailler à l'étranger ou dans les zones rurales du pays où a été livrée la formation, dans les cinq ans suivant la fin de la formation. Nous avons évalué les indicateurs des intentions d'émigration en utilisant des modèles à cotes proportionnelles multivariés.

**Résultats:**

Parmi les répondants, 28% (870/3156) espèrent émigrer à l'étranger, alors que seuls 18% (575/3158) envisagent une carrière en zone rurale. Les étudiants infirmiers désirant pratiquer à l'étranger (rapport des cotes, RC: 1,76; intervalle de confiance à 95%, IC 95%: 1,25–2,48) étaient plus nombreux que les étudiants en médecine. Les souhaits de carrière avant toute installation officielle étaient corrélés avec les intentions actuelles pour les carrières à l'international (RC: 4,49; IC 95%: 3,21–6,29) et dans les zones rurales (RC: 4,84; IC 95%: 3,52–6,66). Le temps passé dans les zones rurales avant installation prédit une préférence pour une carrière rurale (20 contre 0 année: RC: 1,53; IC 95%: 1,19–1,98) et contre le fait de travailler à l'étranger (20 contre 0 année: RC: 0,69; IC 95%: 0,50-0,96).

**Conclusion:**

Une part importante des étudiants interrogés continue à vouloir travailler à l'étranger ou dans les villes après la fin de leur formation. Ces intentions pouvaient être identifiées même avant leur installation. Des normes d'admission tenant compte des années passées dans les zones rurales pourraient favoriser une meilleure rétention des diplômés dans le pays de leur formation et dans les zones rurales.

## Resumen

**Objetivo:**

Evaluar las intenciones de los estudiantes de medicina y enfermería de emigrar al extranjero o trabajar en zonas rurales.

**Métodos:**

Se encuestó a 3199 estudiantes de medicina y enfermería de primer y último curso de 16 instituciones gubernamentales de primer nivel en Bangladesh, Etiopía, India, Kenya, Malawi, Nepal, Tanzania y Zambia. La encuesta incluyó preguntas para identificar los factores que podrían predecir las intenciones de emigrar de los estudiantes. Los resultados primarios fueron las probabilidades de emigrar para trabajar en el extranjero o en zonas rurales en el país de formación en los cinco años posteriores a esta. Los indicadores de las intenciones de migración se evaluaron por medio de modelos de probabilidades proporcionales multivariables.

**Resultados:**

Entre los encuestados, el 28 % (870/3156) esperaba emigrar al extranjero, mientras que solo el 18 % (575/3158) preveía una carrera rural. Más estudiantes de enfermería que de medicina deseaban desarrollar su profesión en extranjero (proporción de probabilidad, OR: 1,76; intervalo de confianza del 95 %, IC: 1,25–2,48). Los deseos sobre la carrera profesional antes de la matriculación se correspondían con los deseos actuales de desarrollar una carrera internacional (OR: 4,49; 95 % IC: 3,21–6,29) y rural (OR: 4,84; 95 % IC: 3,52–6,66). El tiempo pasado en zonas rurales antes de la matriculación predijo la preferencia por una carrera rural (20 frente a 0 años: OR: 1,53; 95 % IC: 1,19–1,98) y en contra de trabajar en el extranjero (20 frente a 0 años: OR: 0,69; 95 % IC: 0,50–0,96).

**Conclusión:**

Una proporción significativa de los estudiantes encuestados todavía tiene la intención de trabajar en el extranjero o en ciudades una vez finalizados los estudios. Dichas intenciones pudieron identificarse incluso antes de la matriculación. Las normas de admisión que contabilizan los años pasados en zonas rurales podrían promover una mayor retención de los graduados en el país de formación y en las zonas rurales.

## ملخص

**الغرض:**

تقييم نوايا طلاب الطب والتمريض للهجرة إلى الخارج أو العمل في المناطق الريفية.

**الطريقة:**

أجرينا دراسة استقصائية عن 3199 طالب من طلاب الطب والتمريض في السنة الأولى والأخيرة في 16 مؤسسة حكومية رائدة في بنغلاديش وإثيوبيا والهند وكينيا وملاوي ونيبال وجمهورية تنزانيا المتحدة وزامبيا. وتضمنت الدراسة الاستقصائية أسئلة لتحديد العوامل التي يمكنها أن تنبئ بنوايا الطلاب للهجرة. وتمثلت الحصائل الأولية في احتمالات الهجرة بغية العمل في الخارج أو العمل في المناطق الريفية في بلد التدريب في غضون خمس سنوات بعد قضاء التدريب. وقمنا بتقييم عوامل التكهن بنوايا الهجرة باستخدام نماذج الاحتمال النسبية متعددة المتغيرات.

**النتائج:**

توقعت نسبة 28 % (870/3156) من المستجيبين الهجرة إلى الخارج، في حين لم يتوقع العمل في المناطق الريفية سوى 18 % فقط (575/3158). ومقارنة بطلاب الطب، رغب المزيد من طلاب التمريض في العمل خارج بلادهم (نسبة الاحتمال: 1.76؛ فاصل الثقة 95 %، فاصل الثقة: 1.25–2.48). وارتبطت رغبات العمل قبل الالتحاق بالتدريب بالنوايا الراهنة في الحصول على وظائف دولية (نسبة الاحتمال: 4.49؛ فاصل الثقة 95 %: من 3.21 إلى 6.29) وريفية (نسبة الاحتمال: 4.84؛ فاصل الثقة 95 %: من 3.52 إلى 6.66). وتنبأ الوقت الذي تم قضاؤه في المناطق الريفية قبل الالتحاق بالتدريب بتفضيل العمل في المناطق الريفية (20 مقابل 0 سنة: نسبة الاحتمال: 1.53، فاصل الثقة: 95 %، فاصل الثقة: من 1.19 إلى 1.98) وعدم العمل في الخارج (20 مقابل 0 سنة: نسبة الاحتمال: 0.69، فاصل الثقة: 95 %، فاصل الثقة: من 0.50 إلى 0.96).

**الاستنتاج:**

ما زالت نسبة كبيرة من الطلاب الذين تم دراستهم استقصائياً ينوون العمل في الخارج أو في المدن بعد قضاء التدريب. ويمكن تحديد هذه النوايا حتى قبل الالتحاق بالتدريب. ويمكن أن تعزز معايير القبول التي تفسر عدد السنوات التي تم قضاؤها في الريف قدراً أكبر من استبقاء الخريجين في بلد التدريب وفي المناطق الريفية.

## 摘要

**目的:**

评估医疗和护理学生出国或在农村地区就业的意向。

**方法:**

我们调查了孟加拉国、埃塞俄比亚、印度、肯尼亚、马拉维、尼泊尔、坦桑尼亚联合共和国和赞比亚16所主要政府院校3199名一年级和毕业班的医疗和护理学生。调查所包含的一些问题被用来确定能够预测学生迁移意向的因素。主要结果是在五年在岗培训期间迁移到国外工作或在培训国农村地区工作的可能性。我们使用多变量比例优势模型评估迁移意向预测因素。

**结果:**

在应答者中，28%（870/3156）期望出国，仅18%（575/3158）期望在乡村工作。较之医疗学生，更多护理学生期望到国外从业（优势比，OR：1.76；95%置信区间，CI：1.25-2.48）。大学入学之前的职业期望与当前国际（OR：4.49；95% CI：3.21-6.29）和乡村（OR：4.84；95% CI：3.52-6.66）就业意向有关联。入学之前在农村地区呆过的时间预示了想到农村就业（20比对0年：OR：1.53，95% CI：1.19-1.98）以及不希望出国工作（20比对0年：OR：0.69，95% CI：0.50-0.96）的程度。

**结论:**

很大一部分受调查的学生仍打算在培训后出国工作或在城市工作。甚至在入学之前就可以分辨出这种意向。在招生标准中列入农村地区呆过的时间，可以将更多的毕业生留在其接受培训的国家和农村地区。

## Резюме

**Цель:**

Оценить стремление студентов, обучающихся на врачей и медсестер, переехать за границу или продолжить практику в сельской местности.

**Методы:**

Мы опросили 3199 студентов первого и последнего курсов, обучающихся на врачей и медсестер в 16 лучших государственных образовательных учреждениях Бангладеш, Эфиопии, Индии, Кении, Малави, Непала, Объединенной Республики Танзании и Замбии. Исследование включало вопросы, помогающие определить факторы, влияющие на желание студентов переехать. Основные результаты определили вероятность переезда за границу или в сельскую местность в стране обучения для работы в течение пятилетнего периода после завершения обучения. Мы оценили факторы, влияющие на стремление переехать, с помощью многоаспектной пропорциональной модели.

**Результаты:**

Среди опрашиваемых лиц 28% (870/3156) планируют переезд за границу и лишь 18% (575/3158) планируют продолжить карьеру в сельской местности. Будущие медсестры чаще, чем будущие врачи, хотят получить работу за границей (коэффициент вероятности: 1,76, 95%; доверительный интервал: 1,25–2,48). Планируемая дальнейшая карьера до поступления в высшее учебное заведение совпадала с текущими стремлениями: для желающих работать за границей (коэффициент вероятности: 4,49, 95%; доверительный интервал: 3,21–6,29) и для желающих работать в сельской местности (коэффициент вероятности: 4,84, 95%; доверительный интервал: 3,52–6,66). Проживание в сельских районах до поступления в высшее учебное заведение часто предопределяло желание работать в сельской местности (20 лет по отношению к 0 лет: коэффициент вероятности: 1,53, 95%; доверительный интервал: 1,19–1,98) по сравнению с желанием работать за границей (20 лет по отношению к 0 лет: коэффициент вероятности: 0,69, 95% доверительный интервал: 0,50–0,96).

**Вывод:**

Значительная часть опрошенных студентов после обучения планируют работать за границей или в городе. Данные цели иногда были определены еще до поступления в высшее учебное заведение. Введение льгот при приеме в учебные заведения может способствовать удержанию выпускников в стране обучения и сельских регионах.

## Introduction

Shortages of physicians and nurses jeopardize health system advances in many low- and middle-income countries (LMIC).^1^^,^^2^ Sub-Saharan Africa has only 2 doctors and 11 nurses or midwives per 10 000 people, compared with approximately 30 physicians and 84 nurses or midwives per 10 000 people in high-income countries.^3^^,^^4^ Emigration of health professionals from LMIC to countries with less need of such professionals exacerbates the global workforce imbalance.^1^^,^^2^^,^^5^ Meanwhile, rural-to-urban migration of those professionals continues to increase provider shortages in rural areas where the need is the greatest.^6^^,^^7^

To address the human resource problems in LMIC’s health sector, the Global Health workforce alliance was formed in 2006 to identify, implement and advocate solutions to the crisis.^8^ Then in 2010, the World Health Organization (WHO) issued a global Code of Practice that intended to curb international migration of health professionals.^9^ WHO has also published recommendations for attracting, recruiting and retaining health workers in rural areas.^6^ Since then, partnerships between governments, institutions and funding organizations have emerged to strengthen LMIC health workforces.^10^^–^^12^ New medical and nursing schools have been established and existing ones expanded,^13^ while curricular reforms have been instituted to enhance graduate retention.^13^^,^^14^

Yet, these investments have been made without systematic analysis of the students’ migration intentions. We know very little regarding the characteristics of students inclined to work in rural areas or remain in the country in which they train.^6^ Without such information, resource-intensive interventions might promote training of graduates with no plans to practice in areas of need. Scholarship-bonding schemes and compulsory service obligations to work in areas with professional shortages struggle to achieve long-term retention,^15^ with most of the health-care professionals leaving shortly after required service terms.^16^ Greater understanding of factors associated with students’ intentions to work in high-demand regions is needed,^6^^,^^17^ and this could help direct admissions’ policies towards selecting individuals most likely to serve in these settings long-term.

Previous studies on health worker retention are mainly from high-income countries^18^^–^^20^ and are not applicable to LMIC. Compared to graduates in high-income countries, LMIC graduates face greater resource disparities between rural and urban settings, as well as the additional lure of providing support to family through remitted salaries earned abroad. Assessments from LMIC are limited in size and fail to compare class years, degree programmes, institutions and countries.^6^ To address this research gap, we conducted a multinational assessment of medical and nursing students’ migration intentions in LMIC by surveying first- and final-year students at leading government institutions (Appendix, available from: http://biostat.mc.vanderbilt.edu/StudentMigration).

## Methods

### Study Design

We considered countries in sub-Saharan Africa and south-east Asia that were classified by WHO as having a critical shortage of health service providers (less than 2.28 physicians, nurses, or midwives per 1000 population).^2^ To avoid confounding effects of language on migration intentions and because most health professional emigration is to English-speaking nations,^21^^,^^22^ we included only countries where English is the language of instruction. To limit the influence of conflict and political turmoil on the results,^22^ we excluded countries with an active United Nations peacekeeping mission or bottom-decile rank in either the Global Peace Index or World Bank Worldwide Governance Indicators.^23^^–^^25^

To enhance similarity between study institution governance structure and founding principles, we excluded countries without both a government medical and nursing school, or in which either school was established after 1993. This date corresponds to a period of increased attention to health sector reform in developing countries that may have affected guiding missions of health training institutions established thereafter.^26^^,^^27^ Three nations meeting selection criteria were excluded due to study resource constraints, leaving eight countries: Bangladesh, Ethiopia, India, Kenya, Malawi, Nepal, the United Republic of Tanzania and Zambia (Appendix). In each country, we selected one government medical school and affiliated government nursing school in the nation’s capital or major commercial city. In countries with multiple qualifying institutions, we used commonly accepted rankings to choose a highly reputed school where institutional approval could also be obtained. Affiliated nursing schools were selected to minimize confounding differences in institutional values, resources and faculty. We focused exclusively on premier government institutions, since they profess a longstanding mission to train future national leaders to address domestic health challenges and employ public funds towards this mission.

The 16 study sites selected are listed in Table 1. Research ethics committee approval was obtained from all sites and from the Vanderbilt University Institutional Review Board.

**Table 1:** Response rates in an eight-country survey of medical and nursing students, 2011–2012

Study site^a^	Respondents/class size^b^ (%)						
	Total		Medical programme			Nursing programme	
			First year	Final year		First year	Final year
Bangladesh	444/538 (83)		152/180 (84)	157/180 (87)		54/80 (68)	81/98 (83)
Ethiopia	438/577 (76)		180/272 (66)	156/178 (88)		52/73 (71)	50/54 (93)
India	434/493 (88)		221/250 (88)	158/180 (88)		34/38 (89)	21/25 (84)
Kenya	634/775 (82)		340/394 (86)	150/225 (67)		78/84 (93)	66/72 (92)
Malawi	394/457 (86)		87/105 (83)	52/59 (88)		169/194 (87)	86/99 (87)
Nepal	203/220 (92)		108/120 (90)	59/63 (94)		19/19 (100)	17/18 (94)
United Republic of Tanzania	355/411 (86)		176/204 (86)	128/149 (86)		38/43 (88)	13/15 (87)
Zambia	297/351 (85)		105/129 (81)	53/65 (82)		108/125 (86)	31/32 (97)
**Total**	**3199/3822 (84)**		**1369/1654 (83)**	**913/1099 (83)**		**552/656 (84)**	**365/413 (88)**
**Total by programme**	**–**		**2282/2753 (83)**			**917/1069 (86)**	

^a^ The 16 study sites included: Dhaka Medical College and Nursing Institute (Bangladesh); Addis Ababa University Schools of Medicine and Nursing (Ethiopia); Maulana Azad Medical College and Ahilya Bai College of Nursing (India); University of Nairobi Schools of Medicine and Nursing Sciences (Kenya); University of Malawi College of Medicine and Kamuzu College of Nursing (Malawi); Tribhuvan University Institute of Medicine and Maharajgunj Nursing Campus (Nepal); Muhimbili University of Health and Allied Sciences Schools of Medicine and Nursing (United Republic of Tanzania); and University of Zambia School of Medicine and its Department of Nursing Sciences (Zambia).

^b^ Class sizes at the time of surveying are as reported by two or more of the following sources for each country: institution registrars, administrators, department heads and student class representatives.

Note: Number of respondents analysed does not include 18 questionnaires discarded due to incompleteness.

### Procedures

We conducted the study from September 2011 to April 2012. Students eligible for our study were first- or final-year students enrolled in medical (Bachelor of Medicine and Surgery; Medical Doctor) or nursing (Bachelor of Science) degree programmes. At each institution, a self-administered questionnaire was given to all eligible students attending a mandatory class lecture. Written informed consent was obtained before survey administration. Survey items assessed student background characteristics such as socioeconomic status and place of origin, attitudes towards rural and international careers and student career intentions (Appendix). Questions, derived from literature review (Appendix), consisted largely of multiple-choice items and five-point Likert scales. Surveys were in English. To minimize potential bias from variable English proficiency, official language translations were provided as an aide alongside the English questions in five countries (Bangladesh, Ethiopia, Malawi, Nepal and the United Republic of Tanzania) where English is not an official language. Translations were validated using independent back-translation. Field testing was performed in each country and country-specific modifications were made to ensure relevant terminology. The two primary outcomes were self-reported likelihood of choosing to work outside the country or in a rural setting inside the country within five years post-training.

Questionnaire results were entered electronically and data entry audited by randomly selecting 22 electronic questionnaires from each country for comparison with hard copies. Audit sample size was calculated to ensure an estimated data entry error rate less than 5% with 95% confidence interval (CI). Questionnaires with more than 25% of responses missing were excluded from analysis.

### Statistical analysis

For each country, we used summary statistics to describe respondent characteristics and multivariable proportional odds models to estimate the independent relationship between 14 selected characteristics (chosen before the analysis) and the likelihood to work internationally or in rural areas. The number of characteristics selected was computed using the smallest country sample size.^28^ The characteristics were: degree programme, class year, sex, number of languages spoken fluently, native language, years spent in a rural setting, maternal tertiary education, first-degree extended family location, self-reported family economic status, expected number of dependents within five years of graduation, post-graduate study plans, pre-matriculation desire for rural or international careers, and country of childhood. Missing values were accounted for using multiple imputation,^29^ and continuous variables were expanded using restricted cubic splines to avoid assumptions of linearity.^28^ Country-specific odds ratios (ORs) were combined using the meta-analysis approach of DerSimonian and Laird,^30^ a random-effects method that adjusts for heterogeneity across institutions. We used R-software version 2.15.1 (http://www.r-project.org) for data analyses.

## Results

At the 16 institutions studied, 3822 students were enrolled in first- or final-year medical or nursing degree classes (Table 1). Of these, 3217 completed the questionnaire, a median class response rate of 87% (range: 66–100%). The primary reason for non-response was student absence during questionnaire administration; six (< 1%) students were present but declined participation. Among collected surveys, 18 (0.6%) were eliminated due to incompleteness and the remaining 3199 were included for analysis. Data entry audits yielded low error rates for all sites (range: 0.02–0.6%).

Ten schools in sub-Saharan Africa comprised 66% (2118/3199) of the study population, while six South Asian schools encompassed 34% (1081/3199) (Table 1). Medical students accounted for 71% (2299/3199) of respondents. Consistent with recent expansions in student enrolment, more first-year students were surveyed than final-year (1369 versus 913). Nearly all students (98%; 3116/3179) were raised in the country of study and most (80%; 2494/3137) reported their families having average or greater wealth (Table 2).

**Table 2:** Characteristics of medical and nursing students in eight low- and middle-income countries, 2011–2012

Characteristic^a^	Overall	Bangladesh	Ethiopia	India	Kenya	Malawi	Nepal	United Republic of Tanzania	Zambia
**Sex, no. (%)^b^**									
Male	1635 (51)	141 (32)	294 (67)	224 (52)	303 (48)	124 (32)	139 (68)	249 (71)	161 (55)
Female	1544 (49)	303 (68)	142 (33)	210 (48)	322 (52)	266 (68)	64 (32)	103 (29)	134 (45)
**Economic Status**,**^c^ no. (%)^b^**									
Poor or below average	643 (20)	100 (23)	112 (26)	20 (5)	83 (14)	147 (38)	21 (10)	85 (24)	75 (26)
Average	1587 (51)	205 (47)	244 (57)	176 (41)	306 (51)	171 (44)	130 (64)	207 (59)	148 (51)
Above average	757 (24)	114 (26)	62 (14)	179 (41)	184 (30)	61 (16)	45 (22)	51 (15)	61 (21)
Wealthy	150 (5)	20 (5)	12 (3)	59 (14)	32 (5)	10 (3)	6 (3)	6 (2)	5 (2)
**Majority of childhood in country of study, no. (%)^b^**	3116 (98)	438 (99)	433 (99)	433 (100)	618 (99)	384 (98)	180 (90)	341 (97)	289 (98)
**Longest time spent in rural area before studies (years), median (IQR)**	0 (0–10)	0 (0–12)	0 (0–10)	0 (0–0)	0 (0–10)	3 (0–10)	1 (0–3)	4 (0–14)	2 (0–10)
**Initial desire (before school) for international career, no. (%)^b^**									
Strongly desired	1140 (36)	200 (46)	159 (37)	78 (18)	245 (39)	162 (42)	51 (25)	124 (35)	121 (41)
Neutral^d^	1657 (52)	201 (46)	223 (52)	286 (66)	323 (51)	184 (47)	121 (60)	188 (53)	131 (44)
Strongly opposed	371 (12)	34 (8)	50 (12)	68 (16)	61 (10)	43 (11)	29 (14)	43 (12)	43 (15)
**Initial desire (before school) for rural career, no. (%)^b^**									
Strongly desired	577 (18)	138 (32)	85 (20)	50 (12)	56 (9)	66 (17)	50 (25)	74 (21)	58 (20)
Neutral^d^	2010 (63)	248 (57)	258 (60)	325 (75)	419 (67)	224 (58)	138 (67)	211 (59)	187 (63)
Strongly opposed	583 (18)	50 (11)	88 (20)	57 (13)	154 (24)	98 (25)	15 (7)	70 (20)	51 (17)

IQR: interquartile range.

^a^ Most relevant variables selected to describe the study population.

^b^ Percentages for each characteristic are computed using the number of students with a non-missing value. The number of missing values is: gender: 20; economic status: 62; majority of childhood in country of study: 20; longest time spent in rural area before studies: 139; initial desire for international career: 31; initial desire for rural career: 29.

^c^ Self-reported economic status of one’s family compared to the rest of the country’s population.

^d^ Combined responses for slightly desired, neutral, and slightly opposed.

Note: The sum of the percentages for each characteristic may not equal 100 due to rounding.

### International career intentions

Of all students, 28% (870/3156) reported being very likely to choose work abroad within five years of completing training (Table 3). International careers were anticipated by 24% (542/2254) of all medical students (26% in sub-Saharan Africa, 362/1406; 21% in South Asia, 180/848) and 36% (328/902) of all nursing students (33% in sub-Saharan Africa, 221/680; 48% in South Asia, 107/222). Only 15% (481/3156) of students reported being very unlikely to migrate. Such students were outnumbered by those expecting to leave in seven of eight countries (Appendix).

**Table 3:** Career intentions within five years after training among medical and nursing students in eight low- and middle-income countries, 2011–2012

Study site	Intention to pursue an international career				Intention to pursue a rural career		
	Very likely, no. (%)^a^	Neutral, no. (%)^a,b^	Very unlikely, no. (%)^a^		Very likely, no. (%)^a^	Neutral, no. (%)^a,b^	Very unlikely, no. (%)^a^
**South Asia**	287 (27)	659 (62)	124 (12)		191 (18)	740 (69)	138 (13)
Medical							
First year	106 (22)	321 (67)	53 (11)		78 (16)	351 (73)	51 (11)
Final year	74 (20)	229 (62)	65 (18)		61 (17)	247 (67)	60 (16)
Total	180 (21)	550 (65)	118 (14)		139 (16)	598 (71)	111 (13)
Nursing							
First year	63 (48)	66 (50)	3 (2)		39 (30)	85 (64)	8 (6)
Final year	44 (49)	43 (48)	3 (3)		13 (15)	57 (64)	19 (21)
Total	107 (48)	109 (49)	6 (3)		52 (24)	142 (64)	27 (12)
**Sub-Saharan Africa**	583 (28)	1146 (55)	357 (17)		384 (18)	1096 (52)	609 (29)
Medical							
First year	243 (28)	499 (57)	131 (15)		150 (17)	498 (57)	226 (26)
Final year	119 (22)	309 (58)	105 (20)		71 (13)	264 (49)	200 (37)
Total	362 (26)	808 (57)	236 (17)		221 (16)	762 (54)	426 (30)
Nursing							
First year	147 (34)	214 (49)	74 (17)		128 (29)	216 (50)	92 (21)
Final year	74 (30)	124 (51)	47 (19)		35 (14)	118 (48)	91 (37)
Total	221 (33)	338 (50)	121 (18)		163 (24)	334 (49)	183 (27)
**Overall**	870 (28)	1805 (57)	481 (15)		575 (18)	1836 (58)	747 (24)
Medical							
First year	349 (26)	820 (61)	184 (14)		228 (17)	849 (63)	277 (20)
Final year	193 (21)	538 (60)	170 (19)		132 (15)	511 (57)	260 (29)
Total	542 (24)	1358 (60)	354 (16)		360 (16)	1360 (60)	537 (24)
Nursing							
First year	210 (37)	280 (49)	77 (14)		167 (29)	301 (53)	100 (18)
Final year	118 (35)	167 (50)	50 (15)		48 (14)	175 (53)	110 (33)
Total	328 (36)	447 (50)	127 (14)		215 (24)	476 (53)	210 (23)

^a^ Percentages are computed using the number of students with a non-missing value. A total of 43 questionnaires lacked responses regarding international migration intentions, while 41 questionnaires were missing responses regarding rural work intentions.

^b^ Combined responses for slightly likely, neutral, and slightly unlikely.

Notes: The sum of the percentages for each study site may not equal 100 due to rounding. Results by country see Appendix, available from: http://biostat.mc.vanderbilt.edu/StudentMigration).

Multivariable analysis showed that nursing students were more likely than medical students to intend careers abroad (OR: 1.76; 95% CI: 1.25–2.48). Final-year students were less likely to plan international careers than first-year counterparts (OR: 0.83; 95% CI: 0.70–0.99; Table 4). The location of a student’s extended family did not correlate with international migration preferences. However, the longer that students had resided in rural settings, the less likely they were to want to work abroad (20 versus 0 years: OR: 0.69; 95% CI: 0.50–0.96; Table 4). Sex, economic status, number of languages spoken, primary language, the mother’s education and number of expected dependents were not independently associated with international migration intentions (Table 4).

**Table 4:** Odds of intending an international or rural career within five years after training among medical and nursing students in eight low- and middle-income countries, 2011–2012

Characteristic	Likelihood of choosing an international career OR (95% CI)	Likelihood of choosing a rural career OR (95% CI)
**Degree programme**		
Medical (ref)	1.00	1.00
Nursing	1.76 (1.25–2.48)	0.96 (0.76–1.22)
**Class year**		
First year (ref)	1.00	1.00
Final year	0.83 (0.70–0.99)	0.67 (0.55–0.82)
**Sex**		
Male (ref)	1.00	1.00
Female	0.90 (0.76–1.07)	0.96 (0.83–1.12)
**No. of languages spoken conversationally**		
2 (ref)	1.00	1.00
3	1.02 (0.82–1.27)	1.09 (0.93–1.28)
**Primary language**		
Non-official (ref)	1.00	1.00
Official	1.00 (0.83–1.22)	1.24 (0.99–1.56)
**Longest time spent in rural area (years)**		
0 (ref)	1.00	1.00
5	0.93 (0.78–1.11)	1.22 (1.02–1.46)
10	0.83 (0.67–1.02)	1.36 (1.11–1.67)
20	0.69 (0.50–0.96)	1.53 (1.19–1.98)
**Mother completed tertiary education**	0.93 (0.72–1.20)	0.90 (0.76–1.06)
**Location of extended family^a^**		
Rural (ref)	1.00	1.00
Semi-urban	0.96 (0.75–1.23)	1.16 (0.91–1.48)
Urban or international	1.04 (0.79–1.37)	0.87 (0.73–1.04)
**Economic status^b^**		
Poor or below average (ref)	1.00	1.00
Average	0.87 (0.68–1.12)	1.07 (0.78–1.47)
Above average	0.82 (0.58–1.17)	1.02 (0.70–1.50)
Wealthy	0.89 (0.48–1.65)	0.97 (0.52–1.83)
**Number of expected dependents after graduation**		
2 (ref)	1.00	1.00
4	1.00 (0.92–1.07)	1.05 (0.97–1.13)
8	0.95 (0.79–1.13)	1.17 (0.98–1.39)
**Likely to pursue post-graduate study**	1.36 (0.96–1.93)	0.92 (0.64–1.32)
**Prematriculation desire for rural career**	0.85 (0.71–1.01)	4.84 (3.52–6.66)
**Prematriculation desire for international career**	4.49 (3.21–6.29)	0.89 (0.77–1.02)
**Lived majority of childhood in country**	0.43 (0.24–0.76)	NA^c^

CI: confidence interval; NA: not applicable; OR: Odds ratio.

^a^ Rural population < 50 000 people, semi-urban population 50 000–200 000 people, urban population > 200 000 people.

^b^ Self-reported economic status of one’s family compared to the rest of the country’s population.

^c^ For the rural career outcome, the characteristic majority of childhood in country was not selected a priori before analysis.

Students who had desired a rural career before matriculating consistently reported having less intention of international migration (OR: 0.85; 95% CI: 0.71–1.01; Table 4), while those who had initially desired careers abroad were far more likely to want to pursue one after graduation (OR: 4.49; 95% CI: 3.21–6.29; Table 4). Final-year students who reported desiring international careers before matriculation remained nearly four times more likely to want to migrate (OR: 3.74; 95% CI: 2.25–6.21; Appendix). The desire for post-graduate specialty education also modestly increased the likelihood of wanting to seek work abroad after training (OR: 1.36; 95% CI: 0.96–1.93; Table 4). Students raised in the country of study desired international careers less than those raised abroad (OR: 0.43; 95% CI: 0.24–0.76; Table 4).

### Rural career intentions

Only 18% (575/3158) of all students reported high likelihood of choosing a rural career within five years of training, including 16% (360/2257) of medical and 24% (215/901) of nursing students (Table 3). In sub-Saharan Africa, students who ruled out a rural career (29%, 609/2089) outnumbered those anticipating one (18%, 384/2089). Medical and nursing students’ intentions of rural work did not differ (OR: 0.96; 95% CI: 0.76–1.22; Table 4). Final-year students were less likely to choose rural careers after training than first-years (OR: 0.67; 95% CI: 0.55–0.82; Table 4). Students who spent longer durations in a rural setting were more likely to anticipate selecting a rural practice (20 versus 0 years: OR: 1.53; 95% CI: 1.19–1.98; Table 4). Sex, economic status, number of languages spoken, primary language, the mother’s education, number of expected dependents and the wish for an international career before starting studies were not independent predictors of rural career intentions.

As with international migration intentions, individuals who upon school entry had desired a rural career were now nearly five times more likely to plan one (OR: 4.84; 95%CI: 3.52–6.66; Table 4). Final-year students who had originally sought rural careers remained over three times more likely to choose one (OR: 3.26, 95% CI: 1.94–5.47; Appendix).

## Discussion

Our data suggest that in nations with critical shortages of health professionals, nearly a quarter of medical students and over a third of nursing students surveyed felt very likely to leave their country within five years post-training. Meanwhile, less than one fifth of students anticipated a rural career. Much of this intent appears suggested by characteristics evident even before enrolment.

Our findings extend data from LMIC that students who have spent significant time in rural settings are more likely to practice in their country and in rural areas.^6^^,^^31^^–^^33^ We found new evidence that this likelihood correlated with the duration of residing in rural areas. Conversely, students raised abroad are most likely to emigrate.^34^ As reported elsewhere,^33^^,^^35^ sex was not independently associated with migration intentions, suggesting that women are making geographic career decisions independent of perceived family obligations.^36^ Confirming other studies,^33^^,^^37^ self-reported economic status did not alone predict desired career location nor did high maternal education.^37^ The association between desire for post-graduate education and intended emigration may be due to the limited supply of specialty training opportunities in many countries studied and indicates the need to strengthen these post-graduate training programmes, while carefully considering which students receive scholarships for additional training.

Although we measured student intentions, our results closely parallel actual rates of migration observed among Asian and African health professionals. Among medical students we surveyed, 26% in sub-Saharan Africa and 21% in South Asia were planning to seek international careers. This resembles school administrative data indicating that 28% of recent sub-Saharan African medical graduates had migrated within five years post-training,^13^ and population estimates suggesting 19–23% of Africa-trained physicians work abroad.^2^^,^^38^ Among South Asian medical graduates – for whom data are limited – 11% work in just four developed countries: Australia, Canada the United Kingdom of Great Britain and Northern Ireland and the United States of America.^5^ Likewise, the 16% of sub-Saharan African medical students planning rural careers in our survey mirrors observed rural practice rates reported by school administrators (16%) and regional population data (13%).^13^^,^^39^ Estimates of nurse migration are more elusive.^36^^,^^40^ Some report 5–11% of all sub-Saharan African nurses (from diploma or degree training programmes) presently practice abroad.^22^ The higher rate of intended international migration that we observed in the same region (32%) likely reflects a greater opportunity among bachelor-degree students to move abroad compared to diploma-only graduates. Similarly, the nursing students we surveyed had lower rural practice intentions (24%) than the observed rural practice rates in sub-Saharan Africa (49%) or South Asia (31%).^39^ This indicates either a stronger urban preference among bachelor-degree nurses or an increasing trend among students to value urban careers more highly than their predecessors.

This study has several strengths. It is large and comprehensive, with 3199 students surveyed from 32 classes in eight countries (Appendix). Our aggregate class response rate is high (84%). Our study is systematic, employing rigid yet relevant selection criteria to identify study sites. All participating nations face significant health worker shortages and ongoing emigration, but possess stable environments where retention policies are not superseded by larger systemic sociopolitical motivators of migration. We independently analysed 14 student characteristics to identify predictors for a career in the country or in rural areas. Our results have implications for education and health-care policy-makers in LMIC and donor nations.

Our sample cannot be generalized to areas where internal conflict or political turmoil may drive migration regardless of student characteristics, nor can it be extrapolated to non-Anglophone countries. Sub-Saharan African countries have comparable physician emigration rates regardless of national language,^22^ indicating that different languages may not impede migrants’ mobility. However, languages do influence the destination that the migrants select,^21^ and possibly rural retention rates of graduates.^13^ The effect of languages on students’ migration plans warrants further research.

Our data suggest that students’ career desires before matriculating may persist into the last year of training. Although our study design cannot exclude recall bias, the direction of recall error is unclear and should be further investigated through longitudinal assessment. Similarly, under-reporting of true migration intentions due to perceived values of the school professionals might create a social bias. However, if such bias is present it would strengthen our results, since the true number of students intending to migrate may be higher and those planning to work in rural areas even lower than we report. Social bias was mitigated by survey anonymity and is unlikely to have affected class years differently. Our cross-sectional questionnaire of students’ intentions is not validated for predicting actual migration behaviour. Nonetheless, the similarity of our results to existing migration statistics suggests that students’ intentions may resemble such behaviour.^2^^,^^13^^,^^22^^,^^38^^,^^39^ Therefore intention could be a feasible measure for migration and does not require long-term follow-up. Formal validation through longitudinal design is warranted.

Further research is needed to understand the migration intentions of students. Our study focused on students at premier public schools, where institutional missions focus on producing practitioners for domestic service and health-care leadership, and where public funds offset educational expenses. Migration ambitions may differ at private or newly-established public institutions with varying school resources, values and admissions standards. Also, longitudinal analysis is needed to discern whether differences in career plans between first- and final-year classes represent an evolution of students’ preferences during the schooling period or a change in class composition resulting from recent enrolment expansions at many institutions studied. Finally, additional migration routes of health professionals in LMIC remain to be studied: from public to private sector and from clinical to administrative sector. This is important research since health professionals’ movements from clinical public-sector work threaten already fragile public-health systems.^1^^,^^2^

Increased demand for health professionals in developed countries is projected to attract even more LMIC graduates,^41^^–^^43^ and is enabled by slow legislative uptake of WHO recommendations on health personnel recruitment.^44^^,^^45^ Given the human resource needs in LMIC, migration between countries and from rural to urban settings deserves attention from policymakers both in countries with health worker shortages and partnering nations that provide technical and financial resources.^46^ Multiple approaches have been recommended by WHO, such as education strategies, regulatory interventions, financial incentives and personal and professional support (Table 5). They must be combined for an effective outcome.^6^ Simply increasing student volume without considering student selection ignores the labour-market dynamics after training,^47^ and may also threaten education quality. Holding graduates in the country or in rural areas with compulsory service schemes does not seem to work in the long term,^15^ since students who migrate leave promptly after such obligations end.^16^ Additional curricular reforms incorporating rural coursework may be inadequate to attract students to rural areas,^6^^,^^19^^,^^20^ as our data suggest short rural exposures have minimal impact compared with selection of students raised in these areas.

**Table 5:** WHO recommendations to improve attraction, recruitment, and retention of health workers in rural areas

Recommendation	Quality^a^ of evidence	Recommendation strength^b^
**Education**		
Target admission of students with rural backgrounds	Moderate	Strong
Locate health training programmes closer to rural areas	Low	Conditional
Expose health students to rural experiences or rotations	Very low	Conditional
Revise health curricula to include rural health topics	Low	Strong
Design continuing education programmes targeted to and accessible by rural health workers	Low	Conditional
**Regulatory interventions**		
Introduce and regulate enhanced scopes of practice in rural areas to promote job satisfaction	Very low	Conditional
Introduce different types of health workers with appropriate training and regulation for rural practice	Low	Conditional
Ensure compulsory service requirements in rural areas are accompanied with support and incentives	Low	Conditional
Tie education subsidies to mandatory rural service	Low	Conditional
**Financial incentives**		
Use bundled incentives (allowances, housing, transport, etc.) to increase financial attractiveness of living in rural areas	Low	Conditional
**Personal and professional support**		
Invest in infrastructure and services to boost living conditions for rural health workers	Low	Strong
Ensure workplace environment is safe and has appropriate equipment, supplies, supervision, and mentorship	Low	Strong
Facilitate interaction between urban and rural health workers	Low	Strong
Design career ladders for rural health workers	Low	Strong
Support exchange of knowledge through rural health professional networks and journals	Low	Strong
Adopt public recognition measures to raise the public profile of rural health workers	Low	Strong

^a^ Quality of evidence rated as high, moderate, low or very low. Data sources derived largely from high-income nations, with inclusion of data from low- and middle-income countries where available.

^b^ Strength of WHO recommendation for each intervention was determined by considering several factors including: quality of evidence, absolute magnitude and durability of effect, balance of advantages versus disadvantages, intensity of resource use required, feasibility globally, and degree of variability in the importance ascribed to the intervention outcome by relevant stakeholders.

Data source: World Health Organization, 2010.^6^

With nearly 64% of people in sub-Saharan Africa and 69% in South Asia residing in rural areas,^3^ it is imperative to boost the long-term attractiveness of rural careers among students or change admissions’ criteria to select students more likely to prefer rural work in the first place. Targeted admissions of rural applicants has been a key component of comprehensive education reforms in developed countries, where schools instituting such policies have increased rural retention from 3–9% to 53–64% of graduates.^48^ However, despite 2010 WHO recommendations supporting such reforms,^6^ all 16 sites we studied admit students based on academic merit, with insufficient consideration of students’ geographic origin. Our data suggest that altering admissions policies in LMIC to favour rural-origin applicants or those desiring to stay in the country will help governments to succeed in retaining health-care professionals where they are most needed, and to avoid spending public and donor resources on training physicians and nurses most likely to leave.

## References

[1] Joint Learning Initiative. Human resources for health: overcoming the crisis. Cambridge: Harvard University; 2004.

[2] The world health report 2006: working together for health. Geneva: World Health Organization; 2006.

[3] World Bank Data, sub-Saharan Africa [Internet]. Washington: World Bank; 2012. Available from: http://data.worldbank.org/region/SSA [cited 2014 Aug 18].

[4] World Bank Data, high income [Internet]. Washington: The World Bank; 2012. Available from: http://data.worldbank.org/income-level/HIC [cited 2014 Aug 18].

[5] Mullan F. The metrics of the physician brain drain. N Engl J Med. 2005;353(17):1810–8. 10.1056/NEJMsa050004 16251537

[6] Increasing access to health workers in remote and rural areas through improved retention: global policy recommendations. Geneva: World Health Organization; 2010. Available from: http://whqlibdoc.who.int/publications/2010/9789241564014_eng.pdf?ua=1 [cited 2014 Jul 10]. 23741785

[7] Lemiere C, Herbst CH, Jahanshahi N, Smith E, Soucat A. Reducing geographic imbalances in health workers in Sub-Saharan Africa [World Bank Working Paper No. 209]. Washington: World Bank; 2011.

[8] Global health workforce alliance. Geneva: World Health Organization: 2014. Available from: http://www.who.int/workforcealliance/about/en/ [cited 2014 Jul 8].

[9] World Health Organization Global Code of Practice on the international recruitment of health personnel. In: Sixty-third World Health Assembly, Geneva, May 2010. Available from: http://www.who.int/hrh/migration/code/practice/en/ [cited 2014 Jan 18].

[10] Collins FS, Glass RI, Whitescarver J, Wakefield M, Goosby EP. Public health. Developing health workforce capacity in Africa. Science. 2010;330(6009):1324–5. 10.1126/science.1199930 21127233 PMC5101931

[11] Mullan F, Frehywot S, Omaswa F, Sewankambo N, Talib Z, Chen C, et al. The Medical Education Partnership Initiative: PEPFAR’s effort to boost health worker education to strengthen health systems. Health Aff (Millwood). 2012;31(7):1561–72. 10.1377/hlthaff.2012.0219 22778346

[12] Eichbaum Q, Nyarango P, Bowa K, Odonkor P, Ferrão J, Mashalla Y, et al. “Global networks, alliances and consortia” in global health education-the case for south-to-south partnerships. J Acquir Immune Defic Syndr. 2012;61(3):263–4. 10.1097/QAI.0b013e31826bf957 22878420

[13] Mullan F, Frehywot S, Omaswa F, Buch E, Chen C, Greysen SR, et al. Medical schools in sub-Saharan Africa. Lancet. 2011;377(9771):1113–21. 10.1016/S0140-6736(10)61961-7 21074256

[14] Buchan J, Couper ID, Tangcharoensathien V, Thepannya K, Jaskiewicz W, Perfilieva G, et al. Early implementation of WHO recommendations for the retention of health workers in remote and rural areas. Bull World Health Organ. 2013;91(11):834–40. 10.2471/BLT.13.119008 24347707 PMC3853959

[15] Frehywot S, Mullan F, Payne PW, Ross H. Compulsory service programmes for recruiting health workers in remote and rural areas: do they work? Bull World Health Organ. 2010;88(5):364–70. 10.2471/BLT.09.071605 20461136 PMC2865657

[16] Tankwanchi ABS, Özden C, Vermund SH. Physician emigration from sub-Saharan Africa to the United States: analysis of the 2011 AMA physician masterfile. PLoS Med. 2013;10(9):e1001513. 10.1371/journal.pmed.1001513 24068894 PMC3775724

[17] Greysen SR, Dovlo D, Olapade-Olaopa EO, Jacobs M, Sewankambo N, Mullan F. Medical education in sub-Saharan Africa: a literature review. Med Educ. 2011;45(10):973–86. 10.1111/j.1365-2923.2011.04039.x 21916938

[18] Lehmann U, Dieleman M, Martineau T. Staffing remote rural areas in middle- and low-income countries: a literature review of attraction and retention. BMC Health Serv Res. 2008;8(1):19. 10.1186/1472-6963-8-19 18215313 PMC2259330

[19] Grobler L, Marais BJ, Mabunda SA, Marindi PN, Reuter H, Volmink J. Interventions for increasing the proportion of health professionals practising in rural and other underserved areas. Cochrane Database Syst Rev. 2009;1(1):CD005314. 19160251 10.1002/14651858.CD005314.pub2

[20] Dolea C, Stormont L, Braichet JM. Evaluated strategies to increase attraction and retention of health workers in remote and rural areas. Bull World Health Organ. 2010;88(5):379–85. 10.2471/BLT.09.070607 20461133 PMC2865654

[21] Adsera A, Pytlikova M. The role of language in shaping international migration [IZA Discussion Paper No. 6333]. Bonn: Institute for the Study of Labor; 2012.

[22] Clemens MA, Pettersson G. New data on African health professionals abroad. Hum Resour Health. 2008;6(1):1. 10.1186/1478-4491-6-1 18186916 PMC2254438

[23] List of peacekeeping operations: 1948–2012. New York: United Nations; 2012. Available from: http://www.un.org/en/peacekeeping/documents/operationslist.pdf [cited 2013 Sep 24].

[24] Global Peace Index: 2011 methodology, results, and findings. Sydney: Institute for Economics and Peace; 2011.

[25] Worldwide governance indicators [Internet]. Washington: World Bank; 2012. Available from: http://info.worldbank.org/governance/wgi/index.aspx#home [cited 2013 Sep 24].

[26] World Bank. World development report 1993: investing in health. New York: Oxford University Press; 1993.

[27] Berman P, Bossert T. A decade of health sector reform in developing countries: what have we learned? In: Data for decision making project symposium; 2000 Mar 15, Washington, United States of America. Boston: Harvard School of Public Health; 2000.

[28] Harrell FEJ. Regression modeling strategies with applications to linear models, logistic regression, and survival analysis. New York: Springer; 2001.

[29] Little RJA, Rubin DB. Statistical analysis with missing data. New York: Wiley; 1987.

[30] DerSimonian R, Laird N. Meta-analysis in clinical trials. Control Clin Trials. 1986;7(3):177–88. 10.1016/0197-2456(86)90046-2 3802833

[31] de Vries E, Reid S. Do South African medical students of rural origin return to rural practice? S Afr Med J. 2003;93(10):789–93. 14652974

[32] Zimmerman M, Shakya R, Pokhrel BM, Eyal N, Rijal BP, Shrestha RN, et al. Medical students’ characteristics as predictors of career practice location: retrospective cohort study tracking graduates of Nepal’s first medical college. BMJ. 2012;345:e4826. 10.1136/bmj.e4826 22893566 PMC3419272

[33] Huntington I, Shrestha S, Reich NG, Hagopian A. Career intentions of medical students in the setting of Nepal’s rapidly expanding private medical education system. Health Policy Plan. 2012;27(5):417–28. 10.1093/heapol/czr052 21880690

[34] Kotha SR, Johnson JC, Galea S, Agyei-Baffour P, Nakua E, Asabir K, et al. Lifecourse factors and likelihood of rural practice and emigration: a survey of Ghanaian medical students. Rural Remote Health. 2012;12:1898. 22967220

[35] Diwan V, Minj C, Chhari N, De Costa A. Indian medical students in public and private sector medical schools: are motivations and career aspirations different? – studies from Madhya Pradesh, India. BMC Med Educ. 2013;13(1):127. 10.1186/1472-6920-13-127 24034988 PMC3851318

[36] Kingma M. Nurses on the move: a global overview. Health Serv Res. 2007;42(3 pt 2):1281–98. 10.1111/j.1475-6773.2007.00711.x 17489915 PMC1955376

[37] Deressa W, Azazh A. Attitudes of undergraduate medical students of Addis Ababa University towards medical practice and migration, Ethiopia. BMC Med Educ. 2012;12(1):68. 10.1186/1472-6920-12-68 22867022 PMC3492144

[38] Docquier F, Bhargava A. A new panel data set on physicians’ emigration rates (1991–2004). Louvain-la-Neuve: Unversité catholique de Louvain. Available from: http://perso.uclouvain.be/frederic.docquier/filePDF/ MBD1_Description.pdf

[39] Global health atlas [Internet]. Geneva: World Health Organization; 2007. Available from: http://apps.who.int/globalatlas/ [cited 2013 Sep 24].

[40] Dovlo D. Migration of nurses from sub-Saharan Africa: a review of issues and challenges. Health Serv Res. 2007;42(3 Pt 2):1373–88. 10.1111/j.1475-6773.2007.00712.x 17489920 PMC1955380

[41] Center for Workforce Studies. The impact of health care reform on the future supply and demand for physicians: updated projections through 2025. Washington: American Association of Medical Colleges; 2010.

[42] Iglehart JK. Reform and the health care workforce–current capacity, future demand. N Engl J Med. 2009;361(19):e38. 10.1056/NEJMp0909521 19846840

[43] Future nursing workforce projections: starting the discussion. London: Center for Workforce Intelligence; 2013.

[44] Siyam A, Zurn P, Rø OC, Gedik G, Ronquillo K, Joan Co C, et al. Monitoring the implementation of the WHO Global Code of Practice on the International Recruitment of Health Personnel. Bull World Health Organ. 2013;91(11):816–23. 10.2471/BLT.13.118778 24347705 PMC3853953

[45] Tankwanchi ABS, Vermund SH, Perkins DD. Has the WHO Global Code of Practice on the international recruitment of health personnel been effective? Lancet Glob Health. 2014;2(7):e390–1. 10.1016/S2214-109X(14)70240-2 25103389

[46] Padilha A, Kasonde J, Mukti G, Crisp N, Takemi K, Buch E. Human resources for universal health coverage: leadership needed. Bull World Health Organ. 2013;91(11):800–800A. 10.2471/BLT.13.118661 24347699 PMC3853947

[47] Sousa A, Scheffler RM, Nyoni J, Boerma T. A comprehensive health labour market framework for universal health coverage. Bull World Health Organ. 2013;91(11):892–4. 10.2471/BLT.13.118927 24347720 PMC3853957

[48] Rabinowitz HK, Diamond JJ, Markham FW, Wortman JR. Medical school programs to increase the rural physician supply: a systematic review and projected impact of widespread replication. Acad Med. 2008;83(3):235–43. 10.1097/ACM.0b013e318163789b 18316867

